# Increasing prevalence of *Coxiella burnetii* seropositive Danish dairy cattle herds

**DOI:** 10.1186/s13028-014-0046-2

**Published:** 2014-07-15

**Authors:** Jens Frederik Agger, Suman Paul

**Affiliations:** 1 Department of Large Animal Sciences, Faculty of Health and Medical Sciences, University of Copenhagen, Groennegaardsvej 8, Frederiksberg DK-1870 C, Denmark; 2 Department of Epidemiology and Public Health, Faculty of Veterinary and Animal Science, Sylhet Agricultural University, Sylhet 3100, Bangladesh

**Keywords:** Coxiella burnetii, Seroprevalence, Herd size, Cattle density, Herd density, Bulk tank milk

## Abstract

A study based on bulk tank milk samples from 120 randomly selected dairy cattle herds was conducted to estimate the prevalence of *Coxiella burnetii* seropositive dairy herds, to describe the geographical distribution, and to identify risk factors. Using the CHEKIT Q-fever Antibody ELISA Test Kit (IDEXX), the study revealed a prevalence of 79.2% seropositive herds, 18.3% seronegative herds, and 2.5% serointermediate herds based on the instructions provided by the manufacturer. Multifactorial logistic regression showed statistically significant associations (*P* < 0.01) between *C. burnetii* seropositivity and increasing herd size (OR = 1.02 per cow increment) and increasing regional average number of cattle per dairy herd (OR = 1.02 per animal increment). Herds >150 cows had 17.9 times higher odds of testing positive compared to herds <80 cows. The regional average number of cattle herds per square kilometer was borderline significantly related to the occurrence of seropositive dairy herds (*P* = 0.06). The results indicate an increased prevalence of seropositive dairy herds since the previous survey in 2008 and an adverse impact of increasing herd size and cattle density on the risk of seropositivity.

## Findings

Repeated surveys of the frequency of infectious diseases are necessary for farmers, agricultural organizations and veterinary services to evaluate the needs for implementing disease control procedures. *Coxiella burnetii*, an obligate intracellular bacterium and a zoonotic agent that may cause Q fever in animals and humans, occurs in cattle almost worldwide [[1],[2]]. The prevalence of *C. burnetii* antibody positive Danish dairy herds in 2008 was 59% [[3]] based on bulk tank milk samples (BTM) from 100 randomly selected herds. Since then publications indicate increasing prevalence in several European countries. Thus, *C. burnetii* infection had been detected in 13 member states of the European Union in 2010 [[4]]. Publications based on BTM samples representative of the target populations of dairy herds reported the prevalence of antibody positive dairy herds to be 79% in the Netherlands [[5]], 38% in the Republic of Ireland [[6]], 65% in Northern Ireland [[7]], 67% in Northern Spain [[8]], and 71% in Wallonia, Belgium [[9]]. Our objectives were therefore, in a repeated study, to estimate the prevalence of *C. burnetii* seropositive dairy herds, to describe the geographical distribution, and to identify risk factors using the herd as the analytical unit based on BTM samples.

In a cross sectional designed survey we randomly selected 120 dairy herds to be tested for the presence of *C. burnetii* antibodies in BTM samples. The sample size was calculated using the formula n = Z^2^pq/l^2^ with an assumed prevalence p = 0.50 and an allowable error on the estimate of l = 0.10 at the 95% confidence level. Although we had a prior knowledge of p = 0.59 [[3]] we used p = 0.50 to maximize the sample size. The calculated sample size was 97 herds. Taking the possibility of losing samples during collection and laboratory handling into account, we decided to include 120 herds. However, no samples were lost. A herd qualified for inclusion in the study if it was delivering milk to a dairy plant at the time of selection in July 2012, and if the herd participated in a milk recording scheme and had all the lactating cows milk yield controlled at least 11 times per year. All 3300 Danish herds, which met the inclusion criteria, were assigned a random number between 0 and 1 (SAS function Ranuni (0)), and the 120 herds with the lowest numbers were included in the study. The samples were tested at the Eurofins Steins Laboratorium A/S Denmark for antibodies against *C. burnetii* using the commercially available CHEKIT Q-fever Antibody ELISA test (IDEXX, Liebefeld-Bern, Switzerland) based on *C. burnetii* inactivated phase 1 and 2 antigens following manufacturer’s instructions. The optical density (OD) of each sample was corrected by subtracting the OD of the negative control. The results were expressed as sample-to-positive values and estimated as S/P = [(OD _sample_ – OD _negative control_)/(OD _positive control_ – OD _negative control_) X 100]. According to the manufacturer, S/P ≥ 40%, S/P < 30% and results in the interval 30% ≤ S/P < 40% were considered as positive, negative and intermediate respectively. However, for the purpose of risk factor analysis in logistic regression we dichotomized the test results as positive for samples with S/P ≥ 40% and as not positive for samples with S/P < 40%. Supplementary herd information for the year 2012 was extracted from the Danish Cattle Database.

The data were analyzed in SAS. The prevalence of positive, negative and intermediate results with confidence interval was estimated using the Proc SURVEYFREQ command. The chi-square (χ^2^) test was used to compare the prevalence found in this study with the prevalence in 2008. Association between herd antibody status and herd size, dominant milk breed type, animal purchase, bulk tank milk somatic cell count, average fat and protein percentage, average milk delivery to a dairy plant per cow, herd type (organic/conventional), regional number of cattle herds per km^2^ (all cattle types) and regional average number of cattle per dairy herd, and regional average number of cattle per cattle herd (all cattle types) were tested by univariable logistic regression followed by multivariable logistic regression with backward elimination of non-significant variables. Statistical significance of the covariates was assessed using the likelihood ratio test based on *P* ≤ 0.05. Collinearity among the selected variables was assessed, and variables with correlation coefficients |ρ| ≤ 0.5 were considered for inclusion in the final model. The values of Hosmer-Lemeshow goodness of fit test were used to validate the final model.

Twenty nine of the 120 BTM samples were tested twice using two separate ELISA plates to validate the precision of the diagnostic test. This was evaluated in a Pearson correlation analysis considering S/P values as measured on a continuous scale and as a categorized variable (S/P ≥ 40 as positive and S/P < 40 as not positive) estimating Kappa (κ) for the agreement between the test results and McNemar’s test.

**Figure 1:**
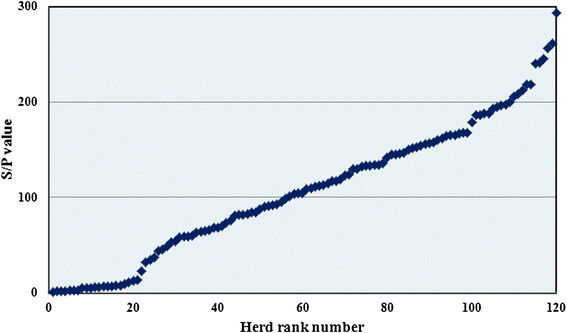
**Array of antibody S/P values to** ***Coxiella burnetii*** **in bulk tank milk samples from 120 randomly selected Danish Dairy herds in July 2012.**

Descriptive analysis showed that an array of the S/P values of the BTM samples ranged from 1 to 293 (Figure 1) with an almost straight line increase (except for the ends of the array) and with no obvious gaps in the test values. This indicates a very dynamic infection status in the population of dairy herds. The apparent prevalences of positive, negative and intermediate herds were 79.2%, 18.3% and 2.5% respectively. Table 1 represents the summary statistics for the three test categories. The prevalence of positive herds in the present study was significantly higher (*P* < 0.01) than the estimate in the study in 2008 [[3]] with 59%, 30% and 11% respectively.

**Table 1:** **Summary statistics of** ***Coxiella burnetii*** **antibody status of 120 randomly selected dairy herds in July 2012**

**Herd category**	**No. of herds**	**Apparent prevalence (95% CI)**	**Mean S/P value ± SE**	**Range of S/P value**
Positive	95	79.17 (71.80; 86.54)	132.80 ± 5.79	44.00 – 293.00
Negative	22	18.33 (11.31; 25.36)	6.82 ± 1.08	1.00 – 23.00
Intermediate	3	2.50 (0.00; 5.33)	34.67 ± 1.45	32.00 – 37.00

**Figure 2:**
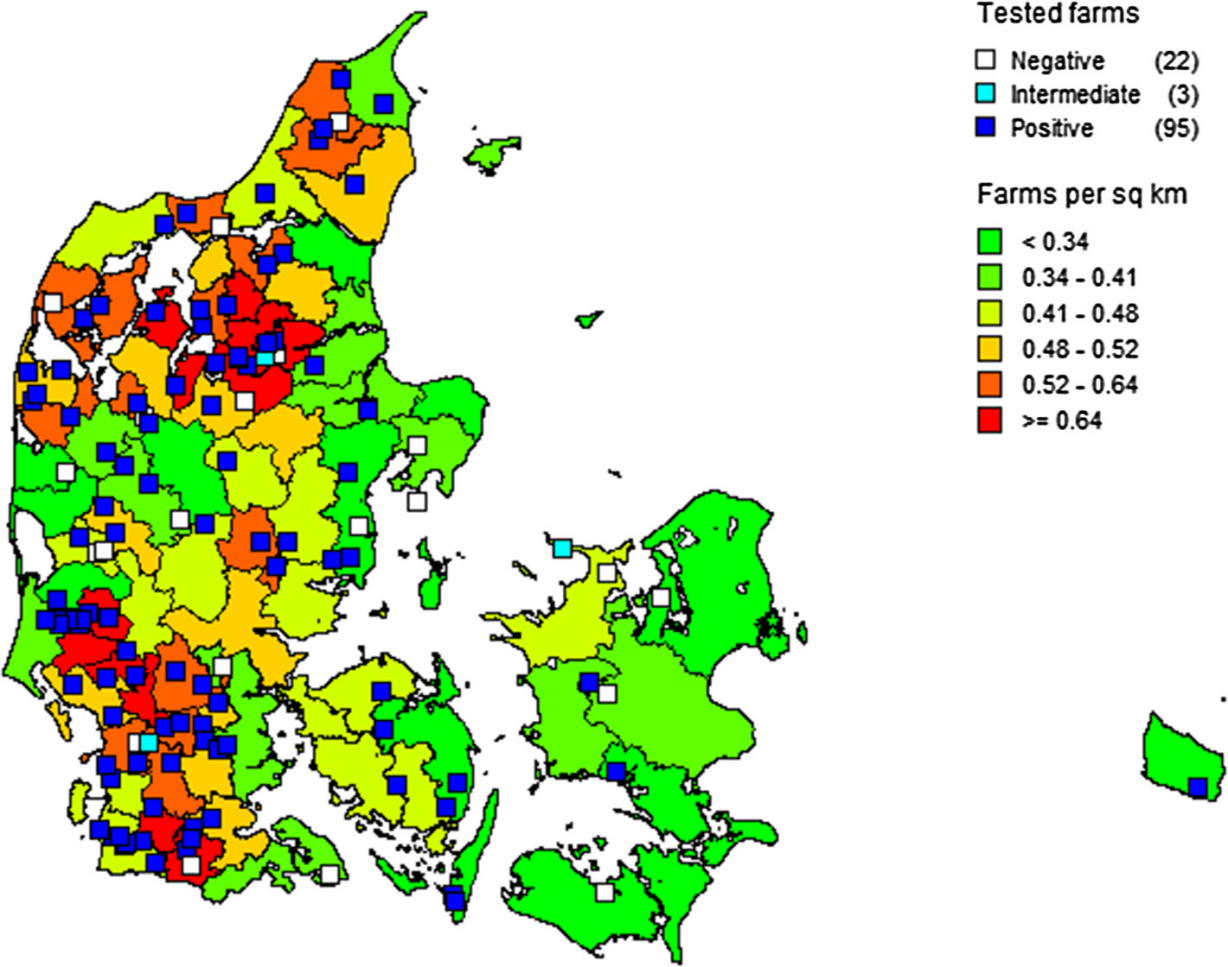
**Regional number of cattle herds per km**^**2**^**(all cattle types) and distribution of 120 randomly selected Danish dairy herds tested for antibodies against** ***Coxiella burnetii*** **in July 2012.**

Herd size, average milk delivery per cow to dairy plant, and regional average number of animals per dairy herd and regional average number of cattle per cattle herd (all cattle types) were analyzed as continuous variables and as categorized into three groups. The variables were found significant in univariable analyses (*P* < 0.05) in both approaches. Regional number of cattle herds per km^2^ (all cattle types) was borderline significant (*P* = 0.06). The final model from multivariable analysis included increasing herd size and increasing regional average number of cattle per dairy herd as continuous scaled variables significantly associated with *C. burnetii* seropositivity (Table 2, Model A). The Hosmer-Lemeshow value confirmed good fit to the data of the final model (*P* = 0.74). The regional number of cattle herds per km^2^ (all cattle types) and the distribution of the sampled herds is presented in Figure 2. The final model from multivariable analysis of the categorized variables only included herd size (Table 2, Model B). The two approaches indicate that the logistic regression model assumption of linearity on the log scale is met.

**Table 2:** **Multivariable logistic regression model of risk factors associated with** ***Coxiella burnetii*** **antibody status (positive or not positive)**

**Model**	**Scale**	**Variable**	**Odds ratio (95% CI)**	***P-value***
A	Continuous	Herd size^a^	1.02 (1.01-1.03)^a^	<0.001
		Regional average number of cattle per dairy herd^a^	1.02 (1.00-1.03)^a^	0.02
B	Categorical	Small herds (<80)	1	<0.001
		Medium herds (80–150)	4.98 (1.39-12.73)	
		Large herds (>150)	17.87 (5.09-31.97)	

^a^Odds ratio calculated per unit change in measurement.

Comparison of duplicate test results of 29 BTM samples in two separate ELISA plates showed a high correlation between the two test results (r^2^ = 0.96). When categorizing the 29 duplicate tests as positive or negative, there was full agreement between the test results (κ =1.00 and *P* = 1). This shows a high precision of the laboratory procedure. This result was expected because the ELISA plates used for the two tests were from the same lot.

The prevalence of 79% *C. burnetii* seropositive Danish dairy herds in 2012 shows an increase compared to the prevalence of 59% in 2008. This corresponds well to an increasing trend in results from other European countries as reviewed above and in reviewed literature in [[3]]. Although exactly the same test kit was used for both studies, it is relevant to consider if this change could be due to drifting of the diagnostic test performance from the laboratory used in the 2008 study compared to the laboratory used in the present study. We therefore had 5 randomly selected samples from the 2012 study tested at both laboratories. There were only very minor deviations in S/P units, which we consider random variation, and there were no differences in the results when categorized into test positive and not positive. We also considered if the increased prevalence of antibody positive herds could be due to the introduction of the Q fever vaccine Coxevac (CEVA) on the EU market in 2011. However, no Danish cattle herds had been vaccinated against Q fever before 2013.

The cattle herd density is highest in the south western and in the north western areas of Denmark. These are also the areas where most of the sampled herds are located. However, there is no clear clustering of herd status compared to density. Garcia-Seco et al. [[10]] did not find clustering of positive herds in a study in the Madrid region, Spain. However, Beaudeau et al. [[11]] in a study of BTM samples from 2600 dairy herds in the region of western France identified some clustering indicating a wind borne impact on the spread of the infection.

Like in our study, Ryan et al. [[6]] and McCaughey et al. [[7]] also found a positive relationship between increasing herd size and test positivity in BTM samples. A recent Danish multilevel study with cow as the analytical unit also found an increasing risk of seropositive cows with increasing herd size [[12]].

The study is based on a random sample of herds, and the results are considered valid for the current prevalence of *C. burnetii* seropositive dairy herds in Denmark. However, as the sample of 120 herds may be slightly too small for more detailed cluster analysis, we have only used simple mapping of the study herds compared to the herd density (Figure 2).

It is concluded that the prevalence of seropositive dairy herds has increased since the previous survey in 2008, and that there is an adverse impact of increasing herd size and of the regional average dairy herd size on the risk of a dairy herd being seropositive.

## Competing interests

The authors declare that they have no competing interests.

## Authors' contributions

Together we designed the study, analysed the data and wrote the manuscript. Both authors read and approved the final manuscript.
